# Glycyrrhizin as a potential disease-modifying therapy for epilepsy: insights into targeting pyroptosis to exert neuroprotective and anticonvulsant effects

**DOI:** 10.3389/fphar.2024.1530735

**Published:** 2025-01-06

**Authors:** Lei Wei, Sijie Ou, Youshi Meng, Lanfeng Sun, Lin Zhang, Yuling Lu, Yuan Wu

**Affiliations:** Department of Neurology, The First Affiliated Hospital of Guangxi Medical University, Nanning, Guangxi, China

**Keywords:** epilepsy, glycyrrhizin, pyroptosis, disease-modifying therapy, neuroinflammation

## Abstract

**Background:**

For patients with epilepsy, antiseizure medication remains the primary treatment; however, it is ineffective in approximately 30% of cases. These patients experience progressive neuronal damage and poor outcomes. Therefore, there is an urgent need for disease-modifying therapy (DMT) that targets the pathogenesis of epilepsy. Glycyrrhizin has shown potential as a DMT in epilepsy due to its multiple targets and diverse mechanisms. Previous studies suggest that glycyrrhizin may regulate key processes involved in epilepsy pathogenesis, such as neuroinflammation and cell death, but its effects on pyroptosis have not been reported.

**Methods:**

This study employed bioinformatics techniques to identify potential molecular targets for glycyrrhizin in epilepsy treatment and then validated using a kainic acid-induced status epilepticus mouse model.

**Results:**

Glycyrrhizin treatment significantly prolonged seizure latency, reduced seizure duration, and alleviated neuronal damage in the status epilepticus mouse model. Molecular experiments indicated that glycyrrhizin may regulate pyroptosis through mediation of the high mobility group box 1 (HMGB1)/Toll-like receptor 4 (TLR4)/nuclear factor kappa-B (NF-κB) signaling pathway.

**Conclusion:**

Glycyrrhizin exerts neuroprotective and anticonvulsant effects in epilepsy by regulating pyroptosis via the HMGB1/TLR4/NF-κB signaling pathway, offering novel insights into its potential as a DMT for epilepsy.

## 1 Introduction

Epilepsy, a chronic neurological disorder characterized by abnormally synchronized neuronal activity in the brain—either localized or widespread—arising from imbalances in excitation and inhibition due to various underlying etiologies, is the second most common neurological disorder after stroke (Fisher et al., 2014; Falco-Walter, 2020). Its recurrent, uncontrollable seizures often impair patients’ ability to work and live normally, and in severe cases, can even leading to death (Shlobin et al., 2024). Globally, epilepsy affects around 50 million people, accounting for approximately 0.5% of the total disease burden (Falco-Walter, 2020). Regardless of whether in low- and middle-income or high-income countries, people with epilepsy face a higher risk of premature death, with the weighted median standardized mortality ratio for epilepsy can reach 2.3–2.6, representing a threefold increase compared to the general population (Levira et al., 2017; Thurman et al., 2017; Secco, 2020). Currently, antiseizure medication (ASM) is the primary treatment for epilepsy; however, conventional ASMs do not alter disease progression or prevent its onset (Pong et al., 2023). In addition, around 30% of epilepsy patients are drug-resistant, with recurrent seizures causing neuronal damage and new epileptogenic foci, thus hastening disease progression (Janmohamed et al., 2020). When diagnosed with drug-resistant epilepsy, second-line treatment options include surgical interventions, dietary modifications, genetic therapies, and other approaches. However, the availability of hospitals equipped to evaluate and perform such treatments is limited. Furthermore, invasive procedures carry additional risks, and not all patients are suitable candidates for surgery. Even after these treatments, patients are typically required to continue taking ASMs (Mesraoua et al., 2023). To address these therapeutic limitations, researchers have recently proposed the concept of DMT for epilepsy, aimed at intervening in the etiology and pathogenesis of epilepsy to slow or reverse its progression (Galanopoulou et al., 2021).

Research on DMT targeting neuroinflammation (Aronica et al., 2017; Ravizza et al., 2024; Lu et al., 2024), oxidative stress (Sandouka et al., 2023; Shekh-Ahmad et al., 2019), glial cells (Çarçak et al., 2023; Vasanthi et al., 2023), programmed cell death (Shi et al., 2024; Pournajaf et al., 2022), RNA (Hansen et al., 2023; Morris et al., 2019), neurodevelopment, and gene therapy (Sciaccaluga et al., 2023; Zhang and Wang, 2021) has shown promising results in animal models or preclinical studies. However, translating these findings to the clinic remains challenging due to the complex pathogenesis of epilepsy and the intricate interrelationships among various therapeutic targets. Consequently, natural compounds with multiple targets and diverse mechanisms may hold greater potential as DMT for epilepsy, and have thus attracted considerable interest.

Glycyrrhizin, a primary active component extracted from *Glycyrrhiza glabra*, is a well-studied natural HMGB1 inhibitor with anti-inflammatory, antioxidant, and immunomodulatory properties (Batiha et al., 2020; Xue et al., 2021). Numerous studies have demonstrated that glycyrrhizin exerts beneficial effects on epilepsy through the modulation of neuroinflammation, oxidative stress, ferroptosis, and mitochondrial autophagy (González-Reyes et al., 2016; Yi et al., 2023; Luo et al., 2023; Paudel et al., 2021; Rosciszewski et al., 2019; Wu et al., 2020), suggesting its potential as a DMT for epilepsy. In addition, it is noteworthy that pyroptosis, a form of programmed cell death that involves inflammatory response (Shi et al., 2017), has been shown to significantly contribute to epilepsy pathogenesis (Zhang et al., 2024; Tan et al., 2015; Hong et al., 2024; Xia et al., 2021). However, whether glycyrrhizin can impact epilepsy by regulating pyroptosis and the potential underlying mechanisms, remains unclear.

Status epilepticus (SE) represents the most severe form of epilepsy, potentially leading to neuronal death and persistent recurrent seizures (Du et al., 2022). The intrahippocampal administration of kainic acid (KA) serves as an established animal model for SE, as it swiftly triggers SE and subsequently advances to epilepsy after a latent phase. This model effectively simulates the pathological characteristics of human temporal lobe epilepsy and is widely utilized for basic epilepsy research and the evaluation of potential therapeutic approaches (Reddy and Kuruba, 2013).

This study employed bioinformatics to investigate how glycyrrhizin influences epilepsy by regulating pyroptosis, and validated these findings in a mouse model, offering new theoretical support for glycyrrhizin as a DMT in epilepsy.

## 2 Materials and methods

### 2.1 Bioinformatics analysis

#### 2.1.1 Candidate target genes of glycyrrhizin in epilepsy treatment

Epilepsy dataset GSE213393 (Organism: *Mus musculus*) was obtained from the Gene Expression Omnibus (GEO) database (https://www.ncbi.nlm.nih.gov/geo/). Differential expression analysis was conducted on the expression matrix of the dataset using the DESeq2 package in R (The R Foundation for Statistical Computing, Vienna, Austria), with screening thresholds set at log2FoldChange >1 and an -log10 (adjusted *P*-value) <2 for differentially expressed genes (DEGs). A volcano plot and heat map of the DEGs were created using the ggplot2 and pheatmap packages in R. Additionally, the GeneCards database (https://www.genecards.org/) was queried with the keyword “glycyrrhizin” to obtain a list of related genes. Candidate target genes for glycyrrhizin in epilepsy were identified by intersecting DEGs with glycyrrhizin-related genes.

#### 2.1.2 Enrichment analysis of glycyrrhizin candidate target genes in epilepsy treatment

Enrichment analyses were performed on candidate target genes to explore the molecular mechanisms by which glycyrrhizin affects epilepsy. Gene Ontology (GO) and Kyoto Encyclopedia of Genes and Genomes (KEGG) enrichment analyses were conducted using the clusterProfiler package in R, considering an adjusted *P*-value <0.01 as the threshold for significant enrichment. Significantly enriched GO terms and KEGG pathways were visualized as bubble or bar plots using the ggplot2 package in R. Gene set enrichment analysis (GSEA) for the pyroptosis pathway was performed using the clusterProfiler package in R. Significant enrichment was determined by criteria of |NES| >1, NOM P-val <0.05, and FDR q-val <0.25. The GSEA results were visualized in R using the enrichplot package, which generated enrichment score curves and rank plots.

#### 2.1.3 Machine learning and diagnostic efficiency evaluation of candidate target genes

Epilepsy dataset GSE73878 (Organism: *Mus musculus*) was downloaded from the GEO database, and gene expression matrix of the dataset was randomly split into a 7:3 ratio for training and testing sets. The training set facilitated model construction and optimization, whereas the testing set served for evaluation and validation. The candidate target genes were incorporated into the training set. Random forest (RF) analysis, utilizing the Boruta and caret packages in R, identified key genes crucial for sample classification, with results visualized via the YSX package. Concurrently, a least absolute shrinkage and selection operator (LASSO) regression classification model was developed on the training dataset using the glmnet package. A support vector machine (SVM) classification model was constructed using the e1071 package. The RF, LASSO regression, and SVM models were validated using the testing set, with confusion matrices calculated at various threshold points. The pROC package in R was utilized to plot receiver operating characteristic (ROC) curves and compute the area under the curve (AUC) from the confusion matrix to assess model performance.

#### 2.1.4 Analysis of protein–protein interaction for candidate target genes

Protein–protein interaction (PPI) analysis was performed on the candidate target genes by importing them into the STRING database (https://string-db.org/), identifying interaction relationships and confidence scores among proteins encoded by the candidate target genes. Using the circlize package in R, a chord diagram was created to visualize interactions between proteins encoded by candidate target genes, with these genes represented as nodes and protein interaction confidence scores as edge weights. Furthermore, topological analysis of the PPI network was conducted using Cytoscape software. Using the maximal clique centrality algorithm, centrality scores were computed for each node, identifying the top 10 highest-scoring genes as hub genes and potential critical regulatory targets.

#### 2.1.5 Prediction of Nlrp3 gene promoter and transcription factor binding sites

The Nlrp3 (NOD-, LRR- and pyrin domain-containing protein 3) gene sequence was obtained from the National Center for Biotechnology Information database, and the promoter region from 2,000 bp upstream to 100 bp downstream of the transcription start site was identified. The promoter sequence FASTA file was downloaded. A search for the NFKB1 transcription factor in the JASPAR database (https://jaspar.elixir.no) identified its binding motif logo. The Nlrp3 promoter sequence was uploaded for binding site prediction, with a relative score >0.8 indicating a high-confidence binding site between the target gene promoter and transcription factor. The Gene Expression Profiling Interactive Analysis (GEPIA) database (http://gepia.cancer-pku.cn/) was utilized to evaluate the expression correlation between NLRP3 and NFKB1 in normal hippocampal tissues. Using the correlation analysis module of GEPIA, NLRP3 and NFKB1 were analyzed for gene expression correlation in normal hippocampal tissue samples from the Genotype-Tissue Expression Project database, resulting in a scatter plot and Pearson correlation coefficient.

### 2.2 Laboratory animals

Male C57BL/6J mice aged 6–8 weeks were sourced from the Experimental Animal Center of Guangxi Medical University. Animals were housed under laboratory conditions with standard temperature, humidity, diet, and a 12-h light/dark cycle. This study received approval from the Animal Care & Welfare Committee of Guangxi Medical University.

### 2.3 Grouping, drug administration protocol, and model establishment

Drugs: Anesthesia was induced using ready-to-use tribromoethanol (M2910; Nanjing Aibei Biotechnology Co., Ltd., Nanjing, China) at a dose of 0.2 mL per 10 g of body weight. KA (HY-N2309; MedChemExpress, Monmouth Junction, NJ, United States) was prepared as a 0.3 μg/μL solution in physiological saline for SE induction. Glycyrrhizin (HY-N0184; MedChemExpress) was prepared in a solution comprising 10% DMSO, 40% PEG300, 5% Tween-80, and 45% saline.

Experimental grouping and drug administration: To determine the optimal dose of glycyrrhizin, twenty-four mice were randomly and equally divided into four groups: model, low-dose, medium-dose and high-dose. The low/medium/high-dose group mice were administered 25/50/100 mg/kg/day glycyrrhizin intraperitoneally, beginning 1 week before model induction. In contrast, the model group mice received intraperitoneal injections of an equivalent volume of the vehicle. The optimal dose for subsequent experiments was selected based on the results from this initial investigation.

In the next phase, twenty-seven mice were randomly assigned to three groups: control, model, and treatment, with each group comprising nine mice. The treatment group received the optimal dose of glycyrrhizin identified in the prior study, beginning 1 week before model induction and continuing until tissue collection, while the model and control groups received intraperitoneal injections of an equivalent volume of the vehicle.

Establishment and evaluation of KA-induced epilepsy model: The KA-induced epilepsy model was established and evaluated by anesthetizing model and treatment group mice with tribromoethanol and securing them in a brain stereotaxic apparatus (RWD Life Science, Shenzhen, China). With the bregma as reference point, 0.3 μg/μL KA was injected into the CA3 region of the right hippocampus with a 5 μL microsyringe under stereotactic guidance (anterior/posterior: −2.46 mm; medial/lateral: 2.75 mm; dorsal/ventral: −2.63 mm) over a period of 3 min. After leaving the injector in place for 3 min post-injection, the scalp was sutured and disinfected. Control group mice received injections of equal volumes of normal saline for sham operation. Epileptic seizures were assessed using the Racine scale (Racine, 1972). Seizure latency was the time from KA injection to the onset of Racine score IV seizures, and seizure duration was the period from the onset to the cessation of these seizures.

### 2.4 Specimen collection and sample handling

One week after model establishment, three randomly selected mice from each group were anesthetized and underwent cardiac perfusion with 4% paraformaldehyde for tissue fixation. The whole brain was extracted and fixed in 4% paraformaldehyde for hematoxylin-eosin (H&E) and Nissl staining. The remaining mice were anesthetized for venous blood collection from the orbital vein for enzyme-linked immunosorbent assay (ELISA). The mice were then sacrificed, and the right hippocampus was dissected for Western blot analysis.

### 2.5 Histological section preparation and staining

Fixed brain tissues were dehydrated in a gradient, infiltrated with paraffin, and embedded to prepare 4-μm thick sections. H&E staining and Nissl staining sections were prepared through dewaxing, rehydration, staining, dehydration, and mounting steps. Hippocampal tissue morphology was examined in stained sections using an optical microscope (Olympus Corporation, Tokyo, Japan). Images of the CA1, CA3, and DG regions were taken to quantify viable cells and neurons.

### 2.6 Western blotting

Hippocampal tissue protein was extracted using RIPA lysis buffer (R0010; Solarbio, Beijing, China) supplemented with PMSF (P0100; Solarbio) and protein phosphatase inhibitor (P1260; Solarbio). The protein concentration was quantified using a BCA protein assay kit (P0010; Beyotime Biotechnology, Nanjing, China) and normalized, following which SDS-PAGE sample loading buffer (WB2001; NCM Biotech, Newport, RI, United States) was added and samples were denatured by boiling. Proteins were separated through SDS-PAGE and transferred onto PVDF membranes using the wet transfer technique. Membranes were blocked with 5% skimmed milk and incubated overnight at 4°C with the following primary antibodies: HMGB1 (1:1,000, Cell Signaling Technology), TLR4 (1:1,000, Abmart), NF-κB p65 (1:1,000, Cell Signaling Technology), phospho-NF-κB p-p65 (1:1,000, Abmart), NLRP3 (1:1,000, Cell Signaling Technology), GSDMD (1:1,000, Abcam), IL-18 (1:1,000, Abmart), and β-actin (1:10,000, Abways). The membrane underwent washing and was incubated with a fluorescent secondary antibody (SA535571; Thermo Fisher Scientific, Waltham, MA, United States) at room temperature for 1 h. Scanning was conducted using the Odyssey infrared fluorescence imaging system (LI-COR Biosciences, Lincoln, NE, United States), followed by grayscale analysis with ImageJ software.

### 2.7 ELISA

IL-18 and IL-1β levels in mouse serum were measured using ELISA kits (MM-0169M1 and MM-0040M1, respectively, Jiangsu Meimian Biotechnology Co., Ltd.). The standards and samples were diluted, added to a microplate, incubated, and then washed. The enzyme-labeled secondary antibody was added, followed by incubation and washing of the microplate. A chromogenic substrate was introduced, and the reaction was subsequently halted with a stop solution. The optical density (OD) of each well was measured at 450 nm using a microplate reader. A standard curve was plotted based on the OD values of the standards and used to determine the concentrations of IL-18 and IL-1β in the samples.

### 2.8 Statistical analysis

SPSS software version 26.0 was used for statistical analyses. Descriptive statistics were performed on all continuous variables, and the Shapiro-Wilk test assessed data distribution normality. Continuous variables with a normal distribution were presented as mean ± standard deviation, and one-way analysis of variance was used for comparisons across multiple groups. A *p*-value less than 0.05 was deemed statistically significant.

## 3 Results

### 3.1 Identification of candidate target genes for glycyrrhizin in epilepsy treatment

**FIGURE 1:**
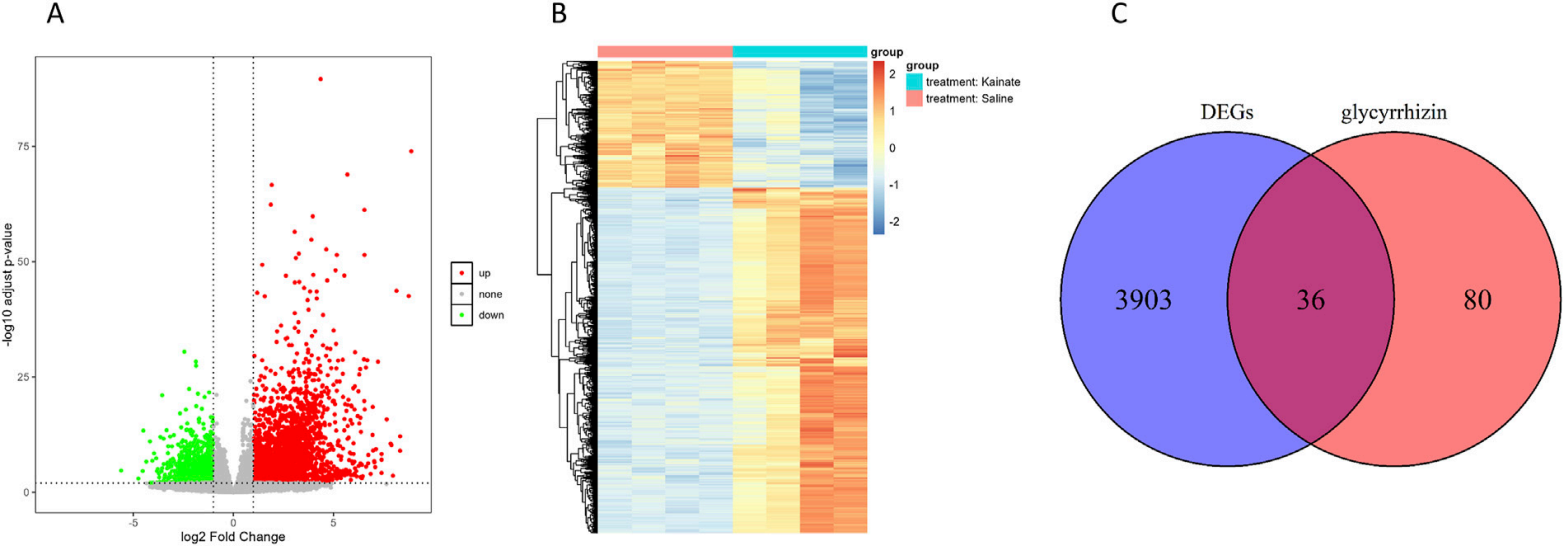
Identification of candidate target genes for glycyrrhizin in epilepsy treatment. **(A)** Volcano plot depicting the statistical significance in DEGs. **(B)** Heat map depicting the expression levels of DEGs across different samples. **(C)** Venn diagram showing the overlap between DEGs and glycyrrhizin-related genes.

Analysis of the GSE213393 dataset revealed 3,939 DEGs, with 2,883 upregulated and 1,056 downregulated (Supplementary Table S1). The volcano plot and heat map of the DEGs were shown in Figures 1A, B, respectively. Subsequently, 116 genes associated with glycyrrhizin were retrieved from the GeneCards database (Supplementary Table S2). Comparing these two gene sets revealed 36 common genes (Figure 1C; Supplementary Table S3), which were considered candidate target genes for glycyrrhizin in epilepsy treatment and further analyzed.

### 3.2 Enrichment analysis of candidate target genes

**FIGURE 2:**
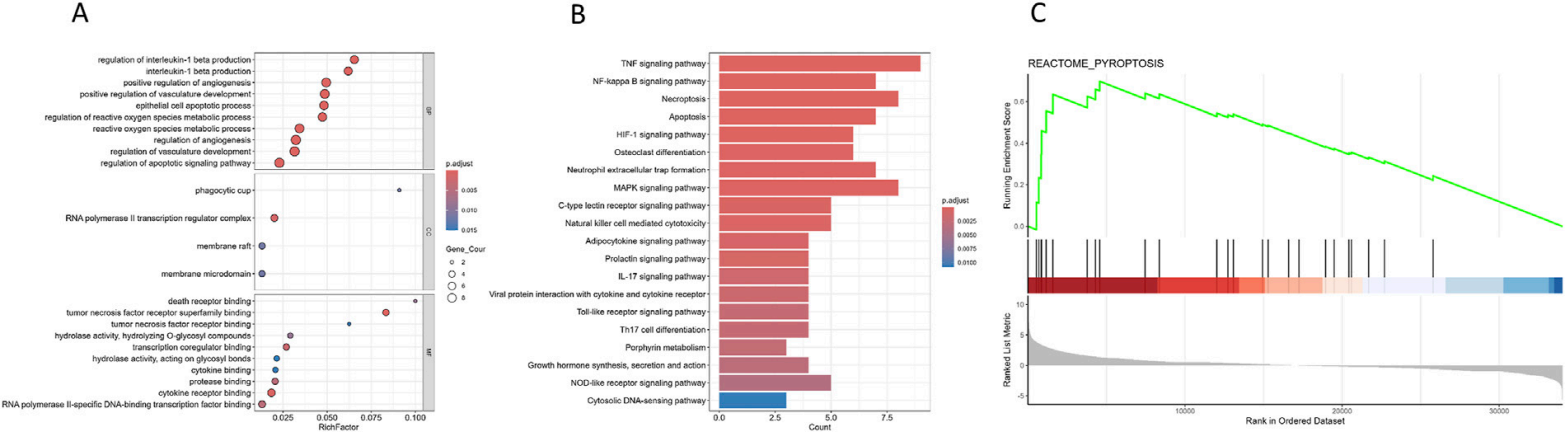
Enrichment analysis of candidate target genes. **(A)** Dot plot representing the results of GO analysis of candidate target genes; the top 10 clusters were selected and displayed according to rich factor ranking. **(B)** Bar plot representing the results of KEGG pathway analysis of candidate target genes; the top 20 pathways were selected and displayed according to p. adjust. **(C)** GSEA enrichment plot for the pyroptosis pathway in GSE213393 dataset (NES = 1.90903, *P* = 0.0002726594, q-value = 0.0014079).

GO and KEGG pathway enrichment analyses revealed significant enrichment of candidate target genes in pathways related to the inflammatory response and programmed cell death, such as “regulation of interleukin-1 beta production,” “TNF signaling pathway,” “NF-κB signaling pathway,” “necroptosis,” and “apoptosis” (Figures 2A, B). Furthermore, GSEA of the pyroptosis pathway demonstrated significant enrichment in the epilepsy group (Figure 2C). The findings indicate that glycyrrhizin’s therapeutic effects in epilepsy may be closely associated with biological processes such as inflammation and programmed cell death.

### 3.3 Machine learning and PPI analysis of candidate target genes

**FIGURE 3:**
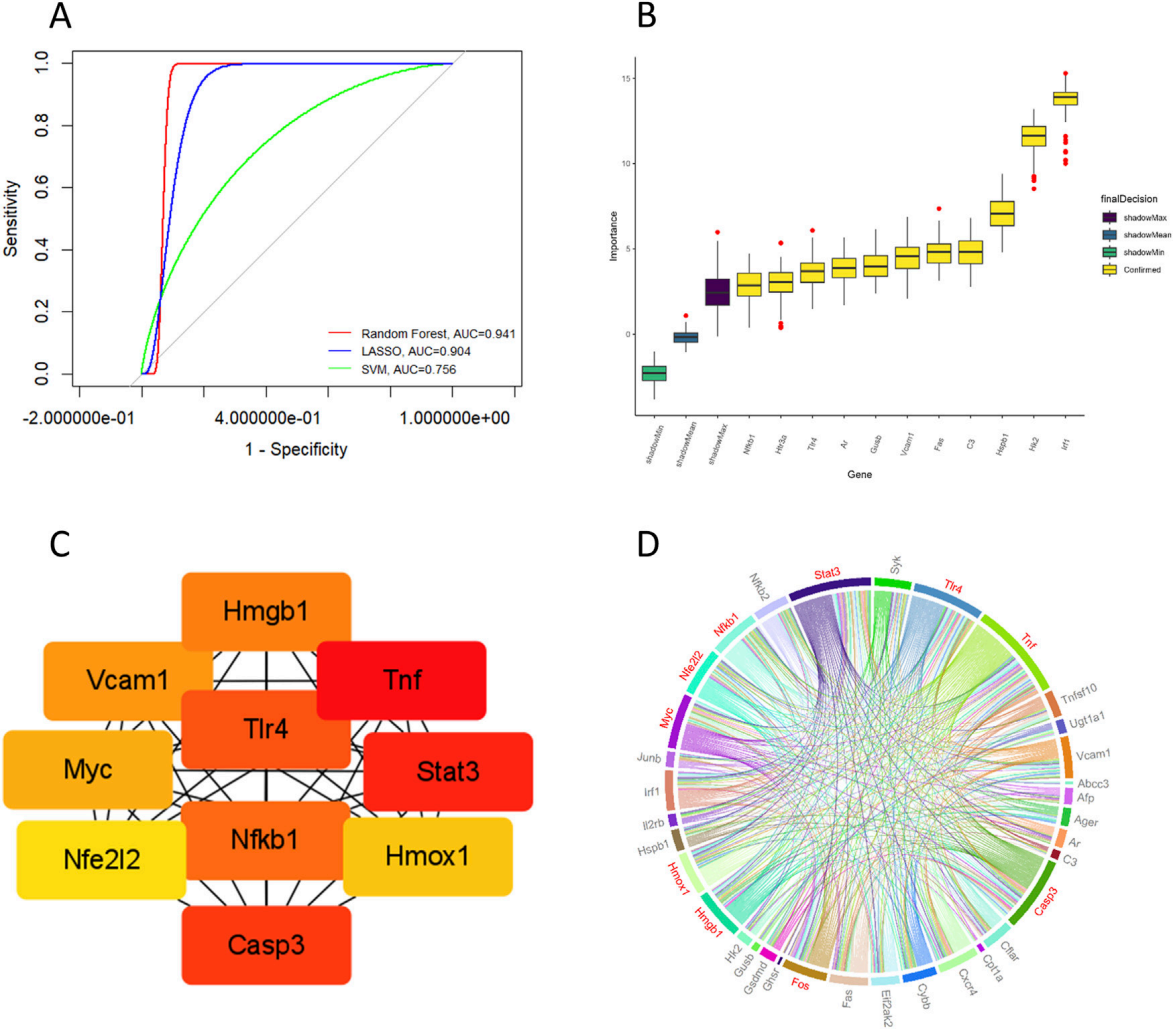
Machine learning and PPI analysis of candidate target genes. **(A)** Through machine learning modeling, ROC curves were plotted for the candidate target genes, with all AUC values > 0.7. **(B)** Key genes were identified using the random forest algorithm. **(C)** Hub genes (Organism: *Mus musculus*) were identified by PPI network analysis. **(D)** A chord diagram shows the interactions between proteins encoded by candidate target genes, with the red sections highlighting the top 10 hub genes.

To further evaluate the diagnostic efficacy of candidate target genes and identify key genes, we used three machine learning algorithms to establish prediction models. Analysis of ROC curves revealed that all models yielded area under the curve (AUC) values exceeding 0.7, indicating that the candidate target genes demonstrated strong diagnostic efficacy in distinguishing between epilepsy and non-epilepsy groups (Figure 3A). The RF algorithm identified 11 key genes among the candidate target genes (Figure 3B). PPI network analysis was conducted to show the interactions between candidate target genes (Figure 3C). A chord diagram was constructed to provide a visual representation of the PPI network, with arc size indicating the significance of candidate genes, and red sections highlighting the top 10 hub genes. The proteins analyzed in downstream Western blotting, such as HMGB1, TLR4, and NF-κB, are included in the hub genes, ranking between 4th and 6th (Figure 3D). Notably, *TLR4* and *NFKB1* were identified as key or core genes by both the RF algorithm and PPI network analysis. HMGB1, a ligand for TLR4, is capable of activating the NF-κB signaling pathway (Chen et al., 2022), suggesting that glycyrrhizin may exert its primary therapeutic action through the regulation of the HMGB1/TLR4/NF-κB pathway.

### 3.4 *NLRP3* gene promoter region has high-confidence binding sites for NFKB1

**FIGURE 4:**
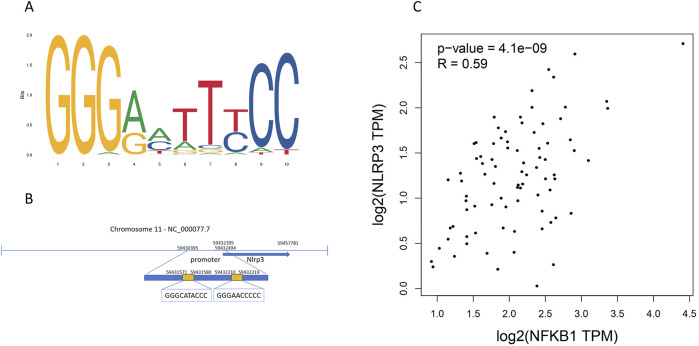
*NLRP3* gene promoter region contains high-confidence binding sites for the NFKB1 transcription factor. **(A)** The binding motif logo of the NFKB1 transcription factor. **(B)** Potential binding sites of NFKB1 in the promoter region of the *NLRP3* gene. **(C)** Correlation scatter plot of *NFKB1* and *NLRP3* gene, with correlation coefficient > 0.5 and *P* < 0.05.

NLRP3 is an important initiator of pyroptosis (Vande Walle and Lamkanfi, 2024). To investigate the transcriptional regulatory mechanism of the *NLRP3* gene, potential binding sites for the transcription factor NFKB1 in the NLRP3 promoter region were analyzed using the JASPAR database. The results indicated that NFKB1 can bind to multiple sites within the NLRP3 promoter region (Figures 4A, B), showing a significant correlation between the two (Figure 4C), suggesting that *NLRP3* gene transcription may be regulated by NF-κB. NLRP3 is essential for pyroptosis, as its activation by transcription factors triggers subsequent pyroptosis pathways.

### 3.5 Glycyrrhizin improves seizures in a KA-Induced SE mouse model

**FIGURE 5:**
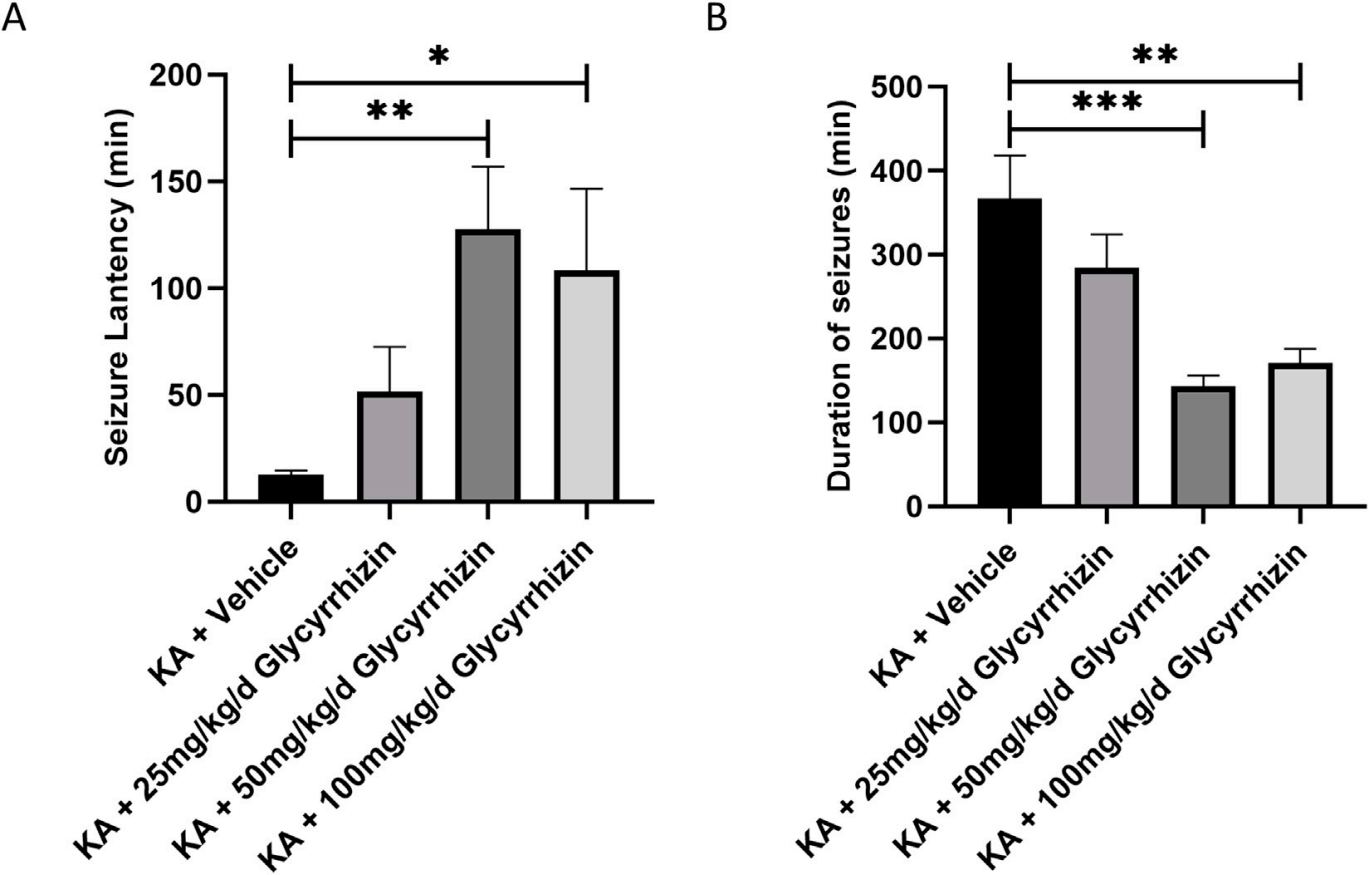
Glycyrrhizin treatment improves seizures in the KA-induced SE mouse model. **(A)** Seizure latency in the mouse model was significantly prolonged by glycyrrhizin treatment in a dose-dependent manner within a certain range. **(B)** Seizure duration in the mouse model was significantly reduced by glycyrrhizin treatment in a dose-dependent manner within a certain range. n = 6 mice/group. *P < 0.05, **P < 0.01, ***P < 0.001.

In the KA-induced SE mouse model, mice that received glycyrrhizin pretreatment exhibited a significantly prolonged seizure latency (Figure 5A, *P* < 0.05 or *P* < 0.01) and significantly reduced seizure duration (Figure 5B, *P* < 0.01 or *P* < 0.001) in a dose-dependent manner within a certain range compared with those of the model group mice. The optimal dose is 50 mg/kg/d, and this dose will be used in subsequent experiments. These results indicate that glycyrrhizin possesses anticonvulsant properties that merit further investigation.

### 3.6 Glycyrrhizin exhibits neuroprotective effects in a KA-Induced SE mouse model

**FIGURE 6:**
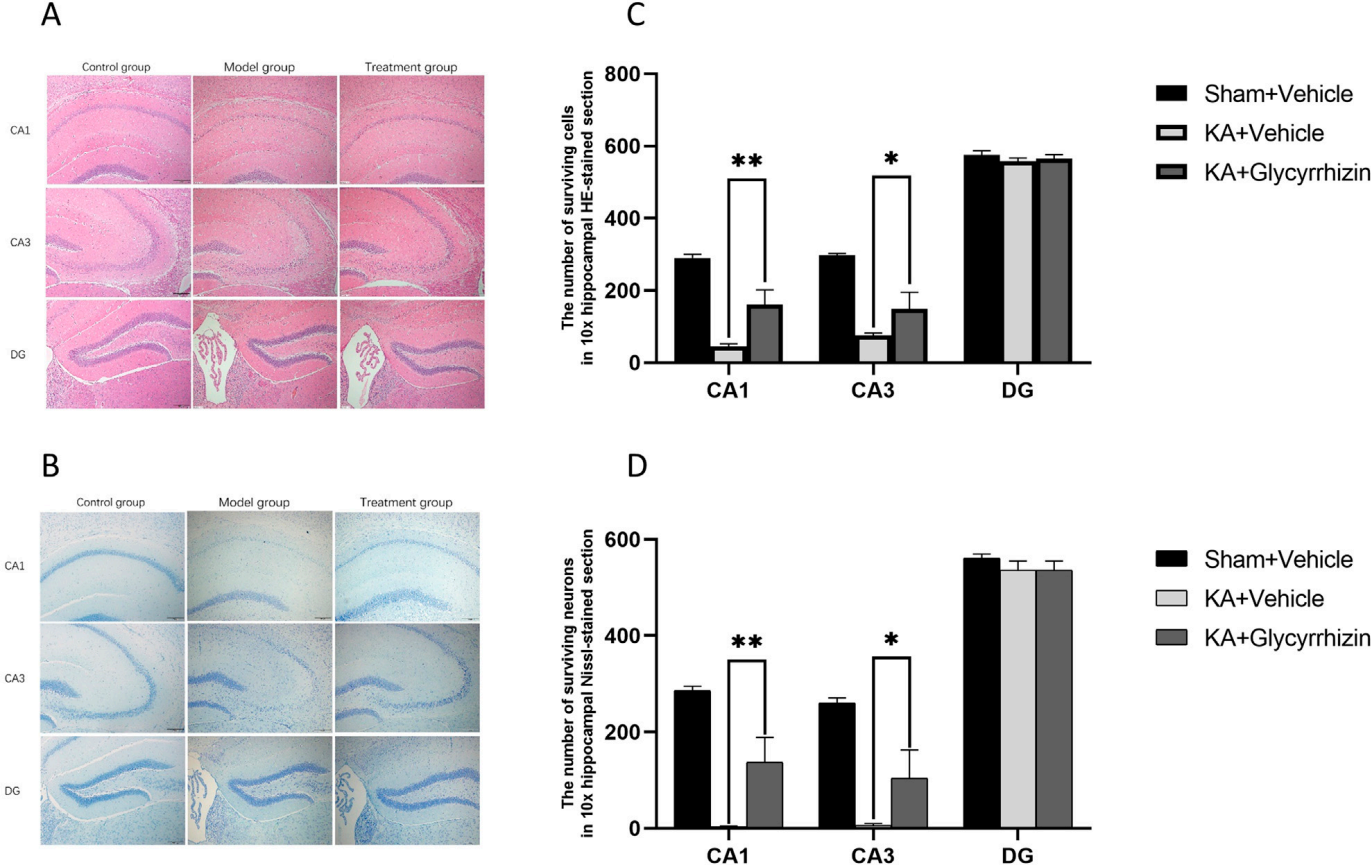
Glycyrrhizin exerts neuroprotective effects in the KA-induced SE mouse model. **(A, B)** H&E and Nissl staining of the hippocampus showed significant neuronal damage in the CA1 and CA3 regions, which was alleviated by glycyrrhizin treatment. **(C, D)** The number of surviving neurons in hippocampal regions in all the groups (n = 3 mice/group). Magnification, ×10. *P < 0.05, **P < 0.01.

H&E and Nissl staining revealed significant neuronal damage in the hippocampal region of model group mice, characterized by decreased neuron numbers, disordered cellular arrangement, and the loss of Nissl bodies. In the glycyrrhizin treatment group mice, these pathological changes were alleviated, with a notable reduction in neuronal death (Figures 6A, B). Quantitative analysis revealed that, compared to model group mice, the number of surviving neurons in the hippocampus of glycyrrhizin treatment group mice was significantly increased (Figures 6C, D, *P* < 0.01 or *P* < 0.05), suggesting that glycyrrhizin exerts a neuroprotective effect.

### 3.7 Potential molecular mechanisms of the neuroprotective and anticonvulsant effects of glycyrrhizin

**FIGURE 7:**
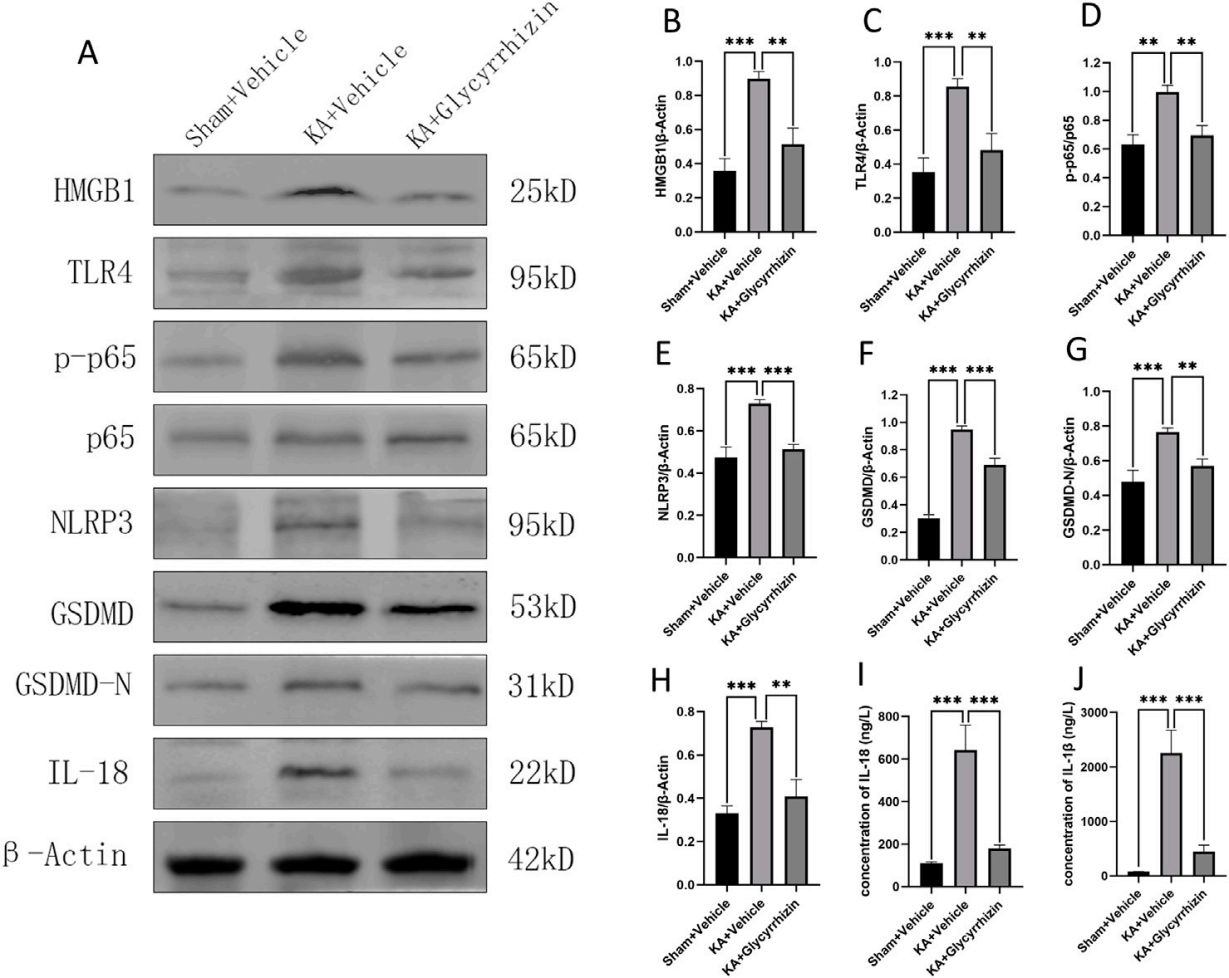
Potential molecular mechanisms underlying the neuroprotective and anticonvulsant effects of glycyrrhizin. **(A)** Western blot images showing bands of protein expression in the hippocampal tissue of each group. **(B–H)** Relative protein expression levels in the hippocampal tissue of each group (n = 6 mice/group). **(I–J)** Inflammatory factor concentrations in the serum of each group (n = 6 mice/group). **P < 0.01, ***P < 0.001.

To explore the molecular mechanisms by which glycyrrhizin affects epilepsy, Western blotting was employed to measure the expression of proteins related to inflammation and pyroptosis in hippocampal tissue, while ELISA was utilized to determine the serum levels of inflammatory factors. Compared to the control group, protein expression levels of HMGB1, TLR4, p-p65, NLRP3, GSDMD, GSDMD-N, and IL-18 were significantly upregulated in the hippocampal tissue of the model group (Figures 7A–H, *P* < 0.01 or *P*< 0.001). This upregulation was significantly reduced by glycyrrhizin treatment (Figures 7A–H, *P* < 0.01 or *P* < 0.001). Furthermore, serum levels of IL-1β and IL-18 were significantly elevated in the model group compared to the control group (Figures 7I, J, *P* < 0.01); this elevation was significantly inhibited by glycyrrhizin treatment (Figures 7I, J, *P* < 0.001), further substantiating the anti-inflammatory properties of glycyrrhizin.

## 4 Discussion

Epilepsy, especially in cases of SE, can lead to permanent nervous system damage. This damage is associated with recurrent seizures as well as long-term consequences, including cognitive impairment, psychiatric disorders, and mortality (Klein et al., 2024a). However, current first-line treatments predominantly rely on ASMs, which are symptomatic treatments that control seizures but cannot modify disease progression or improve long-term outcomes. Despite the introduction of numerous new ASMs over recent decades, the prevalence of drug-resistant epilepsy remains unchanged (Klein et al., 2024b).

The treatment goal for epilepsy should go beyond seizure control, emphasizing the enhancement of patients’ quality of life and their successful reintegration into society (Secco, 2020). To achieve this goal, researchers have introduced the concept of DMTs. These therapies aim to prevent, cure, or modify the progression of epilepsy and its associated comorbidities by addressing the underlying causes and pathogenic mechanisms (Galanopoulou et al., 2021; Klein et al., 2024a). To summarize, the objective is not solely to suppress seizures but to modify the disease trajectory and address its comorbid conditions, ultimately enabling patients with epilepsy to improve their quality of life and successfully reintegrate into society.

This study evaluated glycyrrhizin’s therapeutic efficacy and molecular mechanisms in a SE mouse model. The results show glycyrrhizin significantly enhanced behavioral outcomes and reduced hippocampal neuronal damage. Glycyrrhizin may offer neuroprotection by inhibiting the HMGB1/TLR4/NF-κB signaling pathway, which regulates NLRP3 inflammasome-dependent pyroptosis. This study is the first to propose that glycyrrhizin may affect epilepsy development by modulating pyroptosis, offering a new perspective on its antiepileptic effects.

Glycyrrhizin is a multi-target antiepileptic agent, which affects multiple pathological processes (González-Reyes et al., 2016; Yi et al., 2023). It can alleviate inflammation and cell death, both of which have long-term implications for epilepsy, such as cognitive impairments and psychiatric abnormalities (Mukhtar, 2024; Ngadimon et al., 2024; Henshall and Murphy, 2008). Moreover, inflammation plays a critical role in epilepsy susceptibility (Stredny et al., 2023). The significance of glycyrrhizin lies in its potential to improve epilepsy susceptibility through multiple mechanisms, prevent the onset of epilepsy, and alter the disease trajectory. This could reduce the reliance on ASMs, ultimately enhancing patients’ overall quality of life and social functioning, extending beyond merely controlling seizures.

Glycyrrhizin is widely recognized to inhibit the highly conserved nuclear protein HMGB1. HMGB1 translocates from the nucleus to the extracellular space under various stress conditions, functioning as a damage-associated molecular pattern to modulate early inflammatory and immune responses (Chen et al., 2022). TLR4 is the main receptor for HMGB1, and their interaction activates key transcription factors like NF-κB (Ren et al., 2023).

Pyroptosis is a pro-inflammatory programmed cell death process initiated by the activation of inflammasomes (Fu et al., 2024). NLRP3 is one of the most prominent inflammasomes (Vande Walle and Lamkanfi, 2024). Activation of the NLRP3 inflammasome promotes the maturation of caspase-1, which then cleaves GSDMD. Cleavage of GSDMD releases its N-terminal domain, which forms pores in the cell membrane, thereby promoting the release of IL-18 and IL-1β and triggering an inflammatory response (Shi et al., 2017). Inflammasome activation is regulated by transcription factors (Fu and Wu, 2023), with NF-κB being pivotal in the transcriptional regulation of pro-inflammatory genes (Yu et al., 2020).

In SE models, cell death is a common pathological feature. Previous studies have shown that various types of cell death, including pyroptosis, occur in SE models (Du et al., 2022; Tong et al., 2024). Notably, research has indicated that glycyrrhizin has a protective effect against neuronal death in SE models (Luo et al., 2013); however, its underlying mechanisms remain unclear.

In this study, differential expression analysis identified candidate genes, which were primarily involved in inflammation and programmed cell death. Ten hub genes were identified as key targets within the HMGB1/TLR4/NF-κB signaling pathway.

Potential NF-κB binding sites were then identified in the NLRP3 promoter region. In the SE mouse model, the elevation of HMGB1 expression levels was significantly inhibited by glycyrrhizin treatment, thereby attenuating HMGB1-mediated activation of TLR4 receptors. This led to decreased phosphorylation of the p65 subunit of NF-κB, thereby attenuating NF-κB activation. Reduced NF-κB activity impairs NLRP3 inflammasome assembly and activation, leading to decreased GSDMD expression and activation. This suppression of pyroptosis and inflammatory factor release mitigates neuroinflammation and neuronal damage.

Previous studies align with our findings, indicating that glycyrrhizin modulates inflammation, oxidative stress, and related processes via the HMGB1/TLR4/NF-κB signaling pathway, benefiting neurological disorders such as cerebral ischemia (Li et al., 2020; Yan et al., 2019), cognitive impairment (Kong et al., 2017; Yu M. et al., 2019), and neurotoxicity (Gendy et al., 2023; Tian et al., 2015). Furthermore, glycyrrhizin has been shown to reduce inflammation by regulating pyroptosis through this pathway (Yu R. et al., 2019).

We did not observe spontaneous seizures, cognitive or mental behavioral changes following SE induction. However, glycyrrhizin has been shown to improve long-term outcomes in animal models of epilepsy, including enhanced memory (Paudel et al., 2021), improved learning ability (Webster et al., 2019), and reduced neuronal degeneration (Rosciszewski et al., 2019). Additionally, there were also no positive controls using conventional ASMs. Nonetheless, our study strongly suggests that glycyrrhizin exerts therapeutic effects in the SE mouse model via the regulation of pyroptosis. Additionally, the reduction in neuronal death following glycyrrhizin treatment suggests that glycyrrhizin exerts a neuroprotective effect.

In future studies, we aim to further explore the long-term efficacy of glycyrrhizin in both animal models and human patients. Our focus will include comparing its effectiveness with that of conventional ASMs, optimizing its administration, evaluating appropriate dosing, and assessing its safety and efficacy in patients with epilepsy.

## 5 Conclusion

This study demonstrates that glycyrrhizin regulates pyroptosis via the HMGB1/TLR4/NF-κB signaling pathway, thereby exerting neuroprotective and anticonvulsant effects in the SE mouse model. These preliminary findings, in conjunction with previous studies on the treatment of epilepsy with glycyrrhizin, suggest that glycyrrhizin has disease-modifying effects on epilepsy. This study presents novel evidence supporting glycyrrhizin as a candidate DMT agent for epilepsy.

## Data Availability

The datasets presented in this study can be found in online repositories. The names of the repository/repositories and accession number(s) can be found in the article/Supplementary Material.
